# Polygenic scores as modifiers in Mendelian diseases

**DOI:** 10.1515/medgen-2026-3014

**Published:** 2026-07-08

**Authors:** Henrike O. Heyne, Kerstin U. Ludwig, Axel Schmidt

**Affiliations:** University Hospital Bonn Institute of Human Genetics Venusberg Campus 1 53127 Bonn Germany; University Hospital Bonn Institute of Human Genetics Venusberg Campus 1 53127 Bonn Germany; University of Potsdam Hasso Plattner Institute for Digital Engineering Prof.-Dr.-Helmert-Str. 2–3 14482 Potsdam Germany

**Keywords:** Mendelian diseases, polygenic scores, common variants, incomplete penetrance, variable expressivity

## Abstract

Mendelian diseases are caused by rare pathogenic variants with large effects. However, phenotypes are often different between individuals carrying the same variant (“variable expressivity”), or even absent in some carriers (“incomplete penetrance”), suggesting that the genetic architecture of Mendelian diseases is more complex than previously thought. Common genetic variants are a potential modifier of observed phenotypes. Individual common variants usually have small effects, but in aggregate their effects can be substantial and be quantified as a polygenic score (PGS). The present review summarises available data on how PGS modify phenotypes in Mendelian diseases. We show that modifying effects of PGS on penetrance and expressivity have been observed in carriers of rare pathogenic variants across a broad range of conditions. This suggests that in Mendelian diseases, a modifying effect of the common variant background on penetrance and expressivity might be the rule rather than the exception. We anticipate that increasing data availability and methodological advances will improve our understanding of the genetic architecture of Mendelian diseases, including the joint consideration of both common and rare variants.

## Introduction

Mendelian diseases are characterized by a high heritability driven by one or two genetic variants that are rare in the general population. Phenotypes of most Mendelian diseases show extensive heterogeneity, which is even observed among carriers of the same pathogenic variant within families. For example, the disease can present with different severities, or with a variable age of onset. Within a given Mendelian disease, this is referred to as variable expressivity. In some variant carriers, clinical disease can also be absent, which is termed incomplete penetrance. Incomplete penetrance and variable expressivity have been observed in Mendelian diseases covering almost every organ system – for example, *HFE-*hemochromatosis [1]; hereditary tumour syndromes, such as Lynch syndrome [2]; monogenic forms of epilepsy (e.g. *SCN1A*- or *GABRG2*-related epilepsies; [3]); and some monogenic developmental disorders (e.g. 22q11.2 deletion syndrome, [4]). Explanations for incomplete penetrance and variable expressivity include haplotype-specific effects, unrecognised mosaicism, and environmental factors [5]. However, the genetic background shaped by common genetic variants is present in every individual and might consistently contribute to the observed phenotypes. One of the earliest, notable examples of how common genetic variation can modify a Mendelian phenotype is Huntington’s disease. In 2015, it was shown that in carriers of the established pathogenic *HTT* CAG repeat expansion, age of onset is modified not only by the size of this repeat, but also by genetic variants that are both more frequent in the population (i.e. common variants) and located far away from the *HTT* gene. Specifically, variants at one of the loci identified via genome-wide association studies (GWAS), located near the gene *FAN1* on a different chromosome than *HTT*, accelerate or delay Huntington’s disease onset by 6.1 years and 1.4 years, respectively [6].

Although this example is restricted to the contribution of a single locus, it exemplifies how common variants can modify the effects of rare variants, and that the boundaries between common and rare variants in disease etiologies are not as clear-cut as previously thought. Still, the joint consideration of variants from the entire frequency spectrum has long been neglected, largely driven by an historical division of human genetics into subfields that have studied either the effects of rare variants on rare diseases, or the effects of common variants on common disorders. This dichotomy was enhanced by the fact that different research approaches proved more successful within the respective domains. For example, family-based linkage analyses and, later, exome-wide sequencing within families, have led to the identification of more than 4,000 monogenic disease genes based on rare alleles [7], while in common disorders, case-control studies using array-based genotype data followed by GWAS have identified associations between diseases and tens of thousands of common variants [7]. Notably, the effects of individual common variants on a specific disease or trait are usually small, but can be aggregated and quantified using a polygenic score (PGS).

The aim of this review is to summarise available evidence on the modifying effect of PGS on the phenotypes of Mendelian diseases. We first explain how PGS are generated and introduce the liability threshold model as a theoretical model that can explain the observed data. Next, we present examples of Mendelian diseases covering a broad range of organ systems where a modifying effect of PGS has been observed. Finally, we highlight the current limitations of PGS, and discuss possible future developments in the field of clinical genetics.

## Polygenic scores capture common variant effects

PGS is a neutral term that covers polygenic scores for diseases and non-disease traits, in contrast to the other commonly used term, polygenic risk score (PRS), which refers specifically to disease risk. A PGS is constructed by summing up the effects of up to millions of individual genetic variants into a single score [8]. The main prerequisite for constructing a well-performing (and -powered) PGS is the availability and accessibility of a well-powered GWAS for a disease or trait with relevance to the aetiology of the phenotype of interest. Selection of the GWAS on which the PGS will be based involves specific challenges, which are addressed in the section “Broad application of PGS in rare variant carriers: implementation and challenges”. Approaches to PGS construction vary in terms of the number of genetic factors from the GWAS that are included. Some approaches only include genome-wide significant variants (harbouring common variants with very robust effects on disease risk), while others include the most comprehensive set of all variants in the GWAS dataset. Once generated, the PGS for this specific disease/trait can be calculated in any individual for whom individual-level genetic data are available. In a genetic diagnostic setting, this could be performed via spiking relevant probes into exome- or gene panel sequencing data, extracting genotypes from genome sequencing data, or additional array-based genotyping. The resulting PGS captures the fraction of an individual’s genetic liability for the trait of interest, representing the common variant background. For the purposes of clinical assessment, this individual PGS is then frequently categorized into “low” or “high”, which corresponds to the highest or lowest tertile, quintile, or other percentile of the reference cohort.

## The liability-threshold model of disease

**Figure 1:**
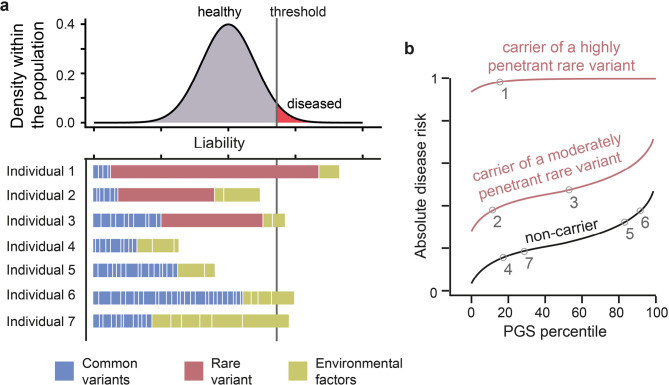
Common and rare variants jointly contribute to disease risk. a) The aetiology of medical conditions can be modelled according to the liability-threshold model. This model suggests that each individual carries a certain “liability” for each disease, and if these liabilities are considered together for each individual across a population, a Gaussian distribution would be observed – with a certain threshold reflecting a clinical cutoff above which a clinical diagnosis is assigned. b) At an individual level, the liability is the combined effect of environmental and genetic factors, with the latter comprising variants across the entire allelic spectrum, including common and rare variants. For example, individuals 1–3 each carry rare variants with fully penetrant (Individual 1) or incompletely penetrant (Individual 2 and 3) effects. Due to the smaller effect size of the rare variant in individuals 2 and 3, environmental or (common) genetic variant effects are necessary to reach the liability threshold. c) The effect of common variants can be measured using polygenic scores (PGS) in individuals with no variants (black line), or variants that are moderately penetrant or highly penetrant (red lines). Common variants frequently contribute to conditions that are caused by rare variants, and penetrance is modified by PGS. Note that PGS percentiles of individuals from a) are indicated in b); also note that PGS captures only a fraction of the common variant effects. Adapted by permission from Springer Nature [51]. This figure is not covered by the CC-BY license of this article; all rights reserved.

The liability-threshold model is a widely used theoretical concept for explaining how binary phenotypes, such as disease occurrence, are caused (**Fig. 1a;** [9]). This model integrates contributions from rare and common genetic variants, as well as environmental factors. The effects of these influences on a given disease are summed up, forming an individual value of disease liability for each individual. If this disease liability exceeds a certain threshold, clinical disease occurs [9]. For some diseases, incidences differ between sexes, and to explain this, sex-specific liability thresholds have been suggested, with infantile hypertrophic pyloric stenosis, which affects five times more males than females, being the most well known example [10]. In classical Mendelian diseases, the effect of a causative rare variant is very high, and would be expected to dominate an individual’s liability profile. Specifically, a fully penetrant variant would be expected to have such a strong effect that the liability threshold would always be exceeded, independent of the contribution of any PGS or environmental factors, and even if the contribution of PGS or environment would be zero (**Fig. 1b,** see individual 1). In contrast, for variants with reduced penetrance, the effect of the variant alone might be insufficient to exceed the liability threshold (**Fig. 1b,** see individuals 2 & 3), with the additional effect of further risk factors being required to trigger clinical disease. These additional effects could be other rare genetic variants, common genetic variants, or environmental factors. This conceptual model was systematically investigated in several studies e.g. a recent UK Biobank study, which showed that individuals with a specific phenotype, but with a low PGS for the respective condition, frequently carried rare damaging variants with potentially larger effect sizes [11].

## PGS as modifier in neurodevelopmental and neurological disorders

Developmental disorders and intellectual disability (DD/ID) are frequently caused by highly penetrant rare variants [12,13]. DD/ID is characterised by genetic heterogeneity, and novel causal rare variants are still being discovered, as driven in part by methodological advances (e.g. long-read sequencing, or extended analyses of non-coding regions [14]). Research has shown that common variants also contribute to DD/ID aetiology [15]. In a recent study of 11,753 patients with rare neurodevelopmental disorders from the Deciphering Developmental Disorders (DDD) study and Genomics England, patients for whom no monogenic diagnosis could be assigned showed significantly reduced PGS for educational attainment and cognitive performance, with 11.2 % of the liability to DD/ID being explained by common variants [12]. Similarly, the UK Biobank cohort was used to investigate the modifying effect of a PGS for educational attainment in carriers of rare, likely damaging variants in 599 dominant DD genes [16]. The analyses showed that the effect of rare variants on cognitive and socioeconomic traits could be partially rescued by a higher PGS for educational attainment. In both studies, rare variants and a PGS for educational attainment contributed in an additive manner to a clinical diagnosis of a developmental disorder.

Similar findings have been reported for other neurodevelopmental disorders. An investigation of autism spectrum disorder (ASD) found that individuals who did not carry a rare variant implicated in ASD risk showed the highest PGS for ASD. In individuals who did carry a rare variant for ASD risk, the PGS for ASD was lower, but still elevated compared to controls [17, 18]. A Mendelian disease that may present as DD/ID or autism is 22q11.2 deletion syndrome. In a study of the effect of common variants on disease expressivity in 962 deletion carriers, PGS for the phenotypes schizophrenia (SCZ) and ID were calculated [19]. Both PGS were partially predictive for the respective phenotype. For example, carriers showed a 33 % risk for SCZ when they were in the high SCZ-PGS group (> 90th percentile), but only 9.1 % SCZ risk when in the low SCZ-PGS group (< 10th percentile).

In monogenic epilepsies, increasing research evidence suggests that penetrance and expressivity are influenced by polygenic background, and this potentially even extends to treatment response [20]. For heterozygous variants previously reported as (likely) pathogenic for epilepsy, penetrance was shown to be modified by a PGS for epilepsy [21]. This finding is in accordance with an earlier independent study of 460 patients with a rare causal variant for developmental and epileptic encephalopathies (DEEs), who had an increased PGS for epilepsy compared to control subjects, with the PGS for epilepsy explaining up to 3.3 % of phenotypic variance [22]. In an investigation of a large family with *GABRG2* p.R82Q related epilepsy, a PGS for epilepsy was increased in family members with a more severe phenotype, which is consistent with the hypothesis that common variants influence clinical expressivity [3].

Finally, PGS have also been investigated in the context of adult-onset monogenic neurological conditions. In *LRRK2*-based Parkinson’s disease, the most common causative variant is p.G2019S, which shows incomplete penetrance. Research has shown that this variant has a significantly increased likelihood to cause disease when in the presence of a high PGS for Parkinson’s disease. Interestingly, the authors found that the effect on penetrance of the PGS for Parkinson’s disease was more pronounced in younger variant carriers [23]. In contrast, a PGS for ischaemic stroke showed no modifying effect on the occurrence of strokes in individuals with monogenic small vessel diseases (CADASIL/*NOTCH3*, n=973 and CARASIL/*HTRA1*, n=546) [24]. The absence of a significant finding may have been attributable to technical issues, e.g. low statistical power; disease-specific issues, such as pathophysiological differences between ischaemic stroke and monogenetic small vessel disease; or a general absence of a modifying effect of common variants in this disorder.

## Hereditary cancer syndromes

In hereditary cancer syndromes, often characterized by a familial clustering of specific tumours that occur at a relatively young age, a modifying influence of PGS has been systematically investigated. Here, we review results for the two most common forms, namely hereditary breast and ovarian cancer, and a form of hereditary colorectal cancer known as Lynch syndrome.

Hereditary breast and ovarian cancer is typically caused by pathogenic, heterozygous variants in the genes *BRCA1* and *BRCA2*. In women, these variants confer absolute risks for breast and ovarian cancer of up to 72 % by the age of 70 years, while in men, they confer absolute risks for prostate cancer of up to 60 % by the age of 85 years, with absolute risks in both sexes differing between carriers of *BRCA1* or *BRCA2* variants [25]. In 2017, data from 15,252 *BRCA1* and 8,211 *BRCA2* mutation carriers showed that breast cancer and ovarian cancer risk was modified by a PGS for breast cancer or ovarian cancer, respectively [26], a finding which has since been replicated in independent studies [2, 27]. In male carriers of pathogenic *BRCA1*/*BRCA2* variants, an association between breast cancer and prostate cancer risk and the respective PGS has also been demonstrated [28].

Besides *BRCA1* and *BRCA2*, breast cancer risk can also be increased, among others, by variants in* PALB2*, *CHEK2*, and *ATM*.* *Due to Finland’s specific population history, variants in the genes* PALB2* (c.1592delT) and *CHEK2* (c.1100delC) have risen to high frequencies in the Finnish population, increasing statistical power for investigating their effects on disease. As demonstrated with data from the FinnGen project [29], the lifetime risk of breast cancer in variant carriers is influenced by a breast cancer specific PGS (*PALB2*: PGS<10th percentile: 49 %, 10th – 90th percentile: 55 %, >90th percentile: 84 %; *CHEK2*: <10th percentile: 9 %, 10th – 90th percentile: 29 %, >90th percentile: 59 %; [30]). Similarly, in the UK Biobank, a breast cancer specific PGS modified the cumulative incidence of breast cancer in carriers of (likely) pathogenic variants in *CHEK2*, *ATM*, and *PALB2* up to the age of 70 years (PGS<10th percentile: 20 %, PGS>90th percentile: 48 %; [27]). Clinical genetic testing for hereditary breast and ovarian cancer is usually performed when the observed phenotype is extreme enough to suggest Mendelian disease. A study from Sweden found that 262 women who underwent such clinical testing had an elevated PGS for breast cancer, even if a causative genetic variant in *BRCA1*, *BRCA2,* or *PALB2* was detected [31]. Thus, common variants appear to contribute to the development of extreme phenotypes that would prompt clinical testing for hereditary breast and ovarian cancer.

Common variants have also been shown to affect penetrance in Lynch syndrome. In two separate studies of individuals from the UK Biobank, the risk of developing colorectal cancer was modified by a PGS for colorectal cancer in 76 and 388 carriers of probable causal variants for Lynch syndrome, respectively [2, 32]. In contrast, this association was not found in two other studies of Lynch syndrome, which applied clinical and family-based recruitment strategies [33, 34]. This discordance was attributed to differences in recruitment, and the higher proportion of highly penetrant genes (*MLH1* and *MSH2*) in clinically recruited individuals [34]. Consistent with this, in highly penetrant genes, PGS modified the penetrance of pathogenic variants to a lesser extent than was the case for moderately penetrant genes in the second UK Biobank study [32].

## Metabolic disorders

Several studies have investigated the extent to which PGS can influence the phenotype of monogenic metabolic disorders. In a study involving 725 patients with familial hypercholesterolemia and evidence of causative variants in the gene *LDLR*, PGS were shown to modify lipid profiles (LDL, HDL, and triglycerides) and risk of cardiovascular or coronary artery disease (CAD). For example, while 24.7 % of individuals in the lowest tertile of the PGS for CAD had cardiovascular disease, this number increased to 40.9 % for patients in the highest tertile [35]. Another study found that on average, a variant causing familial hypercholesterolemia led to a 3.21-fold increased risk of CAD. However, depending on the PGS quintile for CAD, this risk ranged from 1.3-fold (lowest PGS quintile; <20th percentile) to 12.61-fold (highest quintile, i.e. >80th percentile; [2]). The authors of a further study argued that in individuals with familial hypercholesterolemia and an elevated PGS for LDL cholesterol, an undetected monogenic cause is unlikely, which could inform a less aggressive treatment strategy [36].

In a study of individuals diagnosed with monogenic maturity-onset diabetes of the young (MODY), genetically confirmed cases were found to have an elevated PGS for type 2 diabetes (T2D), accounting for 24 % of phenotypic variability. Depending on an individual’s PGS percentile, diabetes risk ranged from 11 % to 81 % in a population cohort of n=424,553. In this study, a PGS for T2D affected not only age-dependent penetrance, but also expressivity (diabetes severity). Similarly, individuals with phenocopies of MODY but no causal variant were found to have a strongly elevated PGS for T2D [37]. Diabetes can also occur in the context of monogenic cystic fibrosis (CF), and this disorder is termed CF-related diabetes. Following a GWAS of 5,740 individuals with CF, a PGS for CF-related diabetes was constructed, and was found to be associated with CF-related diabetes in a replication cohort of 591 individuals with CF [38].

## Other general medical conditions

An impact of PGS on penetrance and/or expressivity has also been reported for other general medical conditions with a known genetic aetiology.

In carriers of rare pathogenic variants for hypertrophic or dilated cardiomyopathy, risk-modifying effects of PGS were demonstrated in data from the UK Biobank. Here, a comparison of the lowest 20 % and the highest 20 % of PGS revealed a 5.7-fold and 2.3-fold increased risk for hypertrophic and dilated cardiomyopathy respectively compared to population baseline [39].

A study examining the risk of developing cardiomyopathy or heart failure following atrial fibrillation found that a PGS for dilated cardiomyopathy predicted this risk independently of either clinical risk factors or the presence of a pathogenic variant in a gene associated with dilated cardiomyopathy (cohorts: UK Biobank and All of Us; [40]). In addition, a rare *SCN5A* variant, p.T220I, which leads to partial loss of function (LoF) of Na_V_1.5, was found to be protective against atrial fibrillation, with carriers having half the risk of the population average. A high PGS for atrial fibrillation (defined as >1 standard deviation above the population average) neutralised the protective effect of the *SCN5A* variant [41].

Common monogenic causes of chronic kidney disease include autosomal dominant polycystic kidney disease (ADPKD) and COL4A-associated nephropathies (including Alport syndrome). Results obtained from the All of Us and UK Biobank biobanks demonstrated that for individuals with one of these presumed monogenic conditions, the risk of chronic kidney disease was modified by a general PGS for chronic kidney disease. For example, the odds ratio for chronic kidney disease in ADPKD variant carriers was 3.03, 35.8, and 54.4 for the lower, middle, and upper PGS tertiles, respectively [42].

The iron storage disease *HFE*-haemochromatosis is a classic example of an autosomal recessive disease in which the main causative variant (p.C282Y in *HFE* gene) shows reduced penetrance. In 2,890 individuals from the UK Biobank who were homozygous for p.C282Y, a PGS for serum iron concentration was significantly associated with the probability of being assigned a diagnosis of haemochromatosis and thus with higher penetrance, but only in males [1]. In addition, disease-specific PGS were associated with their respective disease complications. For example, a PGS for liver cirrhosis was associated with liver cancer diagnoses, an osteoarthritis PGS was associated with osteoarthritis diagnoses, and a PGS for T2D was associated with diabetes [1].

## Ophthalmic disorders

For Mendelian diseases of the visual sensory system, studies of PGS have been limited to date to the most common form of glaucoma, i.e. open-angle glaucoma. Pathogenic variants in *MYOC* account for around 2 – 4 % of all open-angle glaucoma cases, with one of the most prevalent pathogenic variants being p.Q368*. This variant shows incomplete penetrance, as demonstrated by studies performed using the UK Biobank data [43,44]. However, penetrance was significantly influenced by both the PGS for open-angle glaucoma [44], and a PGS that was created using both the clinical diagnosis glaucoma and its ophthalmological correlates (such as increased ocular pressure or alterations in optic nerve morphology; [43]). For example, individuals in the lowest PGS tertile for the latter PGS had an absolute risk of approximately 2 % by the age of 60 years, while for individuals in the highest tertile, the risk increased to around 12 % [43].

## Broad application of PGS in rare variant carriers: implementation and challenges

As illustrated above, extensive evidence shows that common variants, as captured in disease-specific PGS, can modify the phenotypes of a range of Mendelian diseases. Available data suggest that the integration of PGS into the clinical assessment of Mendelian diseases could improve the modelling of the phenotype of individual patients, which includes the explanation of phenomena such as reduced penetrance and variable expressivity.

However, the practical implementation of PGS in Mendelian diseases involves several methodological and clinical challenges [8, 45]. General methodological considerations regarding the construction and investigation of PGS, which have been recently summarised, also apply to individuals carrying pathogenic variants for Mendelian diseases [8]. First, the GWAS used to construct the PGS must be well calibrated, well powered, and able to capture a biological signal that is of aetiological relevance to the Mendelian disease of interest, e.g. a PGS for a tumour disease would not be expected to contribute to the liability for Parkinson’s disease. Second, it is important that the individuals for whom the PGS will be calculated were not included in the GWAS used for PGS calculation [46]. Ideally, the genetic ancestries of individuals included in the GWAS and the individuals in which the PGS will be applied should be similar, since if this is not the case, the performance may be reduced. Specifically, due to the limited representation of individuals with non-European ancestry in GWAS, the prediction performance is often reduced in patients with non-European ancestry, which has triggered discussions on a potential exacerbation of health disparities as PGS become more relevant in clinical practice [47]. Finally, while PGS are designed to directly capture common variant effects, some rare variants might be associated with (or “tagged by”) adjacent common variants that are part of the PGS. This can render the statistical dissection of common and rare variant effects challenging. In situations that require a distinct assessment of these effects, the PGS should be constructed after removal of the rare variant under investigation, including adjacent variants in linkage disequilibrium. Together, these points illustrate that to optimise PGS performance in Mendelian diseases, joint expertise from the two historically separated fields of genetics, i.e. Mendelian and complex diseases is required.

The major prerequisite for integrating rare and common variants is the availability of data from large cohorts, since this enables the effective development and evaluation of statistical models. Fortunately, the number of cohorts with both clinical and genetic data is increasing. While current knowledge on the modifying effects of PGS on rare pathogenic variants is primarily based on retrospective data, ideally, findings and models integrating rare variants with PGS should also be validated in prospective studies. However, for Mendelian diseases this is often unfeasible, due to their low individual incidences. Retrospective cohorts, which offer the potential for longitudinal follow-up data, will therefore remain the mainstay for this research field and for translation into clinical care. However, potential differences between the retrospective cohorts where models are generated and patient cohorts in which the models would be applied, should be considered. Increasing cohort sizes for individuals carrying pathogenic variants will also allow the conduct of GWAS within specific Mendelian diseases, which will facilitate the identification of common variants that contribute to relevant phenotypes, such as age of onset in Huntington’s disease [6].

For hereditary breast and ovarian cancer, an integrated risk prediction model is already being used in clinical care in specific centres. This model takes into account PGS and test results for breast and ovarian cancer risk genes, as well as several other demographic and lifestyle factors (BOADICEA; [48]). To illustrate how PGS can modify risk prediction for a given individual, we calculated cancer risks for a hypothetical 35-year-old female carrier of a pathogenic *BRCA2* variant (see supplemental material for the full set of parameters). In this individual, the predicted 5-year risk for breast cancer was 1.5 %, 4.6 %, and 11.7 % for the 5th, 50th, and 95th breast cancer-specific PGS percentile, respectively. This example demonstrates how in the future, information on common variants, in the form of PGS, could refine estimates of prognosis in patients with Mendelian diseases. Notably, the BOADICEA has been prospectively validated for risk prediction in carriers of pathogenic *BRCA1*/*BRCA2* variants [49].

## Summary and outlook

For multiple Mendelian diseases exhibiting incomplete penetrance or variable expressivity, modifying effects of PGS on the phenotype have been reported. Thus, the genetic architecture of many Mendelian diseases seems to include common variants, which modify the large effects of rare variants. For variants with incomplete penetrance, PGS often have substantial modifying effects on penetrance and expressivity. In contrast, for highly penetrant variants, there is evidence of modifying effects of PGS on expressivity, but the effect on penetrance seems to be low.

In the future, further large cohorts of individuals with rare diseases and genotype and phenotype data will become available, due to efforts such as Genomics England’s National Genomic Research Library [50] and the German “Modellvorhaben Genomsequenzierung” (§ 64e, Fifth Book of the “Sozialgesetzbuch”). These data sets will fuel research into the combined effects of common and rare variants, and facilitate the clinical translation of prediction models that incorporate both common and rare variants.

## Supplementary Material

BOADICEA was accessed using the web interface www.canrisk.org. The following versions were used: BOADICEA model 7.3.2, version 0.6.0; CanRisk v4.0.0. Parameters were set as follows:

Personal Details:

Sex: female, Ethnic group: White, Age: 35 years, Height: 160 cm, Weight: 60kg

Polygenic Scores:

The breast cancer PRS “BCAC 313” was selected. Results were set to three z-scores (–1.6449, 0, and 1.6449), which correspond to the 5th, 50th, and 95th breast cancer-specific PGS percentile, respectively.

Family History:

To indicate the presence of a pathogenic variant in *BRCA2*, within the Gene Tests section of the index patient, the “BRCA2 test” was set to “direct” with the result “Mut-present”.

All other parameters were set to default.

## References

[1] Pilling LC, Atkins JL, Melzer D Genetic modifiers of penetrance to liver endpoints in HFE hemochromatosis: Associations in a large community cohort. Hepatology. Baltimore, Md.; 2022;76.

[2] Fahed AC, Wang M, Homburger JR, Patel AP, Bick AG, Neben CL Polygenic background modifies penetrance of monogenic variants for tier 1 genomic conditions. Nat Commun. 2020;11.

[3] Oliver KL, Scheffer IE, Ellis CA, Grinton BE, Berkovic SF Investigating the effect of polygenic background on epilepsy phenotype in “monogenic” families. EBioMedicine. 2024;109.

[4] McDonald-McGinn DM, Hain HS, Emanuel BS, Zackai EH (1993). 22q11.2 Deletion Syndrome. GeneReviews®.

[5] Kingdom R, Wright CF Incomplete Penetrance and Variable Expressivity: From Clinical Studies to Population Cohorts. Front Genet. 2022;13.

[6] Identification of Genetic Factors that Modify Clinical Onset of Huntington’s Disease. Cell. 2015;162.

[7] Claussnitzer M, Cho JH, Collins R, Cox NJ, Dermitzakis ET, Hurles ME A brief history of human disease genetics. Nature. 2020;577.

[8] Choi SW, Mak TS-H, O’Reilly PF Tutorial: a guide to performing polygenic risk score analyses. Nat Protoc. 2020;15.

[9] Falconer DS The inheritance of liability to certain diseases, estimated from the incidence among relatives. Ann Hum Genet. 1965;29.

[10] Carter CO, Evans KA Inheritance of congenital pyloric stenosis. J Med Genet. 1969;6.

[11] Baya NA, Lassen FH, Hill B, Venkatesh SS, Currant H, Lindgren CM (2025). Individuals whose phenotype deviates from genetic expectation defined by common variation are enriched for rare. Individuals whose phenotype deviates from genetic expectation defined by common variation are enriched for rare.

[12] Huang QQ, Wigdor EM, Malawsky DS, Campbell P, Samocha KE, Chundru VK Examining the role of common variants in rare neurodevelopmental conditions. Nature. 2024;636.

[13] Jansen S, Vissers LELM, de Vries BBA The Genetics of Intellectual Disability. Brain Sci. 2023;13.

[14] Chen Y, Dawes R, Kim HC, Ljungdahl A, Stenton SL, Walker S De novo variants in the RNU4-2 snRNA cause a frequent neurodevelopmental syndrome. Nature. 2024;632.

[15] Niemi MEK, Martin HC, Rice DL, Gallone G, Gordon S, Kelemen M Common genetic variants contribute to risk of rare severe neurodevelopmental disorders. Nature. 2018;562.

[16] Kingdom R, Beaumont RN, Wood AR, Weedon MN, Wright CF Genetic modifiers of rare variants in monogenic developmental disorder loci. Nat Genet. 2024;56.

[17] Klei L, McClain LL, Mahjani B, Panayidou K, De Rubeis S, Grahnat A-CS How rare and common risk variation jointly affect liability for autism spectrum disorder. Mol Autism. 2021;12.

[18] Weiner DJ, Wigdor EM, Ripke S, Walters RK, Kosmicki JA, Grove J Polygenic transmission disequilibrium confirms that common and rare variation act additively to create risk for autism spectrum disorders. Nat Genet. 2017;49.

[19] Davies RW, Fiksinski AM, Breetvelt EJ, Williams NM, Hooper SR, Monfeuga T Using common genetic variation to examine phenotypic expression and risk prediction in 22q11.2 deletion syndrome. Nat Med. 2020;26.

[20] Piet MM, Braun KPJ, Koeleman BPC, Stevelink R Monogenic no more: are all epilepsies polygenic?. Trends Genet TIG. 2026;S0168-9525(26)00005-3.

[21] Stevelink R, Piet MM, Bos Y, van ’t Slot R, Braun KPJ, Koeleman BPC (2025). Penetrance of pathogenic epilepsy variants is low and shaped by common genetic background. Epilepsia.

[22] Campbell C, Leu C, Feng Y-CA, Wolking S, Moreau C, Ellis C The role of common genetic variation in presumed monogenic epilepsies. EBioMedicine. 2022;81.

[23] Iwaki H, Blauwendraat C, Makarious MB, Bandrés-Ciga S, Leonard HL, Gibbs JR Penetrance of Parkinson’s Disease in LRRK2 p.G2019S Carriers Is Modified by a Polygenic Risk Score. Mov Disord Off J Mov Disord Soc. 2020;35.

[24] Cho BPH, Harshfield EL, Al-Thani M, Tozer DJ, Bell S, Markus HS Association of Vascular Risk Factors and Genetic Factors With Penetrance of Variants Causing Monogenic Stroke. JAMA Neurol. 2022;79.

[25] Petrucelli N, Daly MB, Pal T (1993). BRCA1- and BRCA2-Associated Hereditary Breast and Ovarian Cancer. GeneReviews®.

[26] Kuchenbaecker KB, McGuffog L, Barrowdale D, Lee A, Soucy P, Dennis J Evaluation of Polygenic Risk Scores for Breast and Ovarian Cancer Risk Prediction in BRCA1 and BRCA2 Mutation Carriers. J Natl Cancer Inst. 2017;109.

[27] Hassanin E, May P, Aldisi R, Spier I, Forstner AJ, Nöthen MM Breast and prostate cancer risk: The interplay of polygenic risk, rare pathogenic germline variants, and family history. Genet Med Off J Am Coll Med Genet. 2022;24.

[28] Lecarpentier J, Silvestri V, Kuchenbaecker KB, Barrowdale D, Dennis J, McGuffog L Prediction of Breast and Prostate Cancer Risks in Male BRCA1 and BRCA2 Mutation Carriers Using Polygenic Risk Scores. J Clin Oncol Off J Am Soc Clin Oncol. 2017;35.

[29] Kurki MI, Karjalainen J, Palta P, Sipilä TP, Kristiansson K, Donner KM FinnGen provides genetic insights from a well-phenotyped isolated population. Nature. 2023;613.

[30] Mars N, Widén E, Kerminen S, Meretoja T, Pirinen M, Della Briotta Parolo P The role of polygenic risk and susceptibility genes in breast cancer over the course of life. Nat Commun. 2020;11.

[31] Kvist A, Kämpe A, Törngren T, Tesi B, Baliakas P, Borg Å Polygenic scores in Familial breast cancer cases with and without pathogenic variants and the risk of contralateral breast cancer. Breast Cancer Res BCR. 2025;27.

[32] Hassanin E, Spier I, Bobbili DR, Aldisi R, Klinkhammer H, David F Clinically relevant combined effect of polygenic background, rare pathogenic germline variants, and family history on colorectal cancer incidence. BMC Med Genomics. 2023;16.

[33] Jenkins MA, Buchanan DD, Lai J, Makalic E, Dite GS, Win AK Assessment of a Polygenic Risk Score for Colorectal Cancer to Predict Risk of Lynch Syndrome Colorectal Cancer. JNCI Cancer Spectr. 2021;5.

[34] Dueñas N, Klinkhammer H, Bonifaci N, Spier I, Mayr A, Hassanin E Ability of a polygenic risk score to refine colorectal cancer risk in Lynch syndrome. J Med Genet. 2023;60.

[35] Paquette M, Chong M, Thériault S, Dufour R, Paré G, Baass A Polygenic risk score predicts prevalence of cardiovascular disease in patients with familial hypercholesterolemia. J Clin Lipidol. 2017;11.

[36] Futema M, Bourbon M, Williams M, Humphries SE Clinical utility of the polygenic LDL-C SNP score in familial hypercholesterolemia. Atherosclerosis. 2018;277.

[37] Murray Leech J, Beaumont RN, Arni AM, Chundru VK, Sharp LN, Colclough K Common genetic variants modify disease risk and clinical presentation in monogenic diabetes. Nat Metab. 2025;7.

[38] Aksit MA, Pace RG, Vecchio-Pagán B, Ling H, Rommens JM, Boelle P-Y Genetic Modifiers of Cystic Fibrosis-Related Diabetes Have Extensive Overlap With Type 2 Diabetes and Related Traits. J
Clin Endocrinol Metab. 2020;105.

[39] Klasfeld SJ, Knutson KA, Miller MR, Fauman EB, Berghout J, Moccia R Common genetic modifiers influence cardiomyopathy susceptibility among the carriers of rare pathogenic variants. HGG Adv. 2025;6.

[40] Wijdeveld LFJM, Ajufo E, Challa SP, Rämö JT, Wang X, Kany S Cardiomyopathy-Associated Gene Variants in Atrial Fibrillation. JAMA Cardiol. 2025;10.

[41] Wanner JS, Krafft M, Niiranen T, Zimmerman DS, Ellinor PT Leveraging a Genetic Proxy to Investigate the Effects of Lifelong Cardiac Sodium Channel Blockade. Circulation. 2025;152.

[42] Khan A, Shang N, Nestor JG, Weng C, Hripcsak G, Harris PC Polygenic risk alters the penetrance of monogenic kidney disease. Nat Commun. 2023;14.

[43] Craig JE, Han X, Qassim A, Hassall M, Cooke Bailey JN, Kinzy TG Multitrait analysis of glaucoma identifies new risk loci and enables polygenic prediction of disease susceptibility and progression. Nat Genet. 2020;52.

[44] Zebardast N, Sekimitsu S, Wang J, Elze T, Gharahkhani P, Cole BS Characteristics of p.Gln368Ter Myocilin Variant and Influence of Polygenic Risk on Glaucoma Penetrance in the UK Biobank. Ophthalmology. 2021;128.

[45] Martínez-Minguet D, Noel R, G Simón A, Pastor Ó Challenges in clinical translation of polygenic risk score analyses: A systematic review. Genet Med Off J Am Coll Med Genet. 2026;28.

[46] Ellis CA, Oliver KL, Harris RV, Ottman R, Scheffer IE, Mefford HC Inflation of polygenic risk scores caused by sample overlap and relatedness: Examples of a major risk of bias. Am J Hum Genet. 2024;111.

[47] Martin AR, Kanai M, Kamatani Y, Okada Y, Neale BM, Daly MJ Clinical use of current polygenic risk scores may exacerbate health disparities. Nat Genet. 2019;51.

[48] Lee A, Mavaddat N, Wilcox AN, Cunningham AP, Carver T, Hartley S BOADICEA: a comprehensive breast cancer risk prediction model incorporating genetic and nongenetic risk factors. Genet Med Off J Am Coll Med Genet. 2019;21.

[49] Yang X, Mooij TM, Leslie G, Ficorella L, Andrieu N, Kast K Validation of the BOADICEA model in a prospective cohort of BRCA1/2 pathogenic variant carriers. J Med Genet. 2024;61.

[50] Smedley D, Smith KR, Martin A, Thomas EA, McDonagh EM 100,000 Genomes Pilot on Rare-Disease Diagnosis in Health Care – Preliminary Report. N Engl J Med. 2021;385.

[51] Breen G Common genetic variants contribute more to rare diseases than previously thought. Nature. 2024;636.

